# Signatures of Breast Cancer Progression in the Blood: What Could Be Learned from Circulating Tumor Cell Transcriptomes

**DOI:** 10.3390/cancers14225668

**Published:** 2022-11-18

**Authors:** Emanuela Fina

**Affiliations:** Humanitas Research Center, IRCCS Humanitas Research Hospital, Via Manzoni 56, 20089 Milan, Italy; emanuela.fina@humanitasresearch.it or emanuela1.fina@gmail.com

**Keywords:** circulating tumor cells, breast cancer, gene signatures, biomarkers, metastasis

## Abstract

**Simple Summary:**

Breast cancer is a disease characterized by abnormal cell growth. Breast cancer cells can spread outside the breast through blood vessels and lymph vessels. Early detection of breast cancer cells circulating in the blood is of utmost importance for informing disease status and molecular features.

**Abstract:**

Gene expression profiling has revolutionized our understanding of cancer biology, showing an unprecedented ability to impact patient management especially in breast cancer. The vast majority of breast cancer gene expression signatures derive from the analysis of the tumor bulk, an experimental approach that limits the possibility to dissect breast cancer heterogeneity thoroughly and might miss the message hidden in biologically and clinically relevant cell populations. During disease progression or upon selective pressures, cancer cells undergo continuous transcriptional changes, which inevitably affect tumor heterogeneity, response to therapy and tendency to disseminate. Therefore, metastasis-associated signatures and transcriptome-wide gene expression measurement at single-cell resolution hold great promise for the future of breast cancer clinical care. Seen from this perspective, transcriptomics of circulating tumor cells (CTCs) represent an attractive opportunity to bridge the knowledge gap and develop novel biomarkers. This review summarizes the current state-of-the-science on CTC gene expression analysis in breast cancer, addresses technical and clinical issues related to the application of CTC-derived signatures, and discusses potential research directions.

## 1. Introduction

Breast cancer is the most frequent neoplasm among women worldwide, accounting for about 30% of female cancers [1] and showing increases of 3.1% per year, a trend that is likely to continue due to population growth and ageing [2]. Randomized clinical trials have provided evidence that screening with mammography significantly reduces mortality from breast cancer by a relative risk of 20% [3,4]. Early diagnosis actually improves the chance of survival since cancer that is contained in the breast or that has spread only to regional lymph nodes can be cured with a high success rate. However, almost 30% of patients with breast cancer who are free of disease after initial local and regional treatments experience tumor recurrence months or decades later [5,6,7]. Major obstacles to the effectiveness of therapeutic protocols are (i) cellular and molecular tumor heterogeneity, which affects response to systemic treatments [8] and makes patients stratification or outcome prediction difficult [9], and (ii) metastatic growth, which implies the dissemination of cancer cells through the circulatory system and the invasion of distant vital organs and tissues, mainly bone, lung, brain and liver [10]. Lower indeed is the 5-year survival rate of patients with metastases (about 27%) compared to women with the diagnosis of early stage disease (about 90%) [11,12]. Therefore, although treated with the aim to prolong survival, stage IV is not considered curable with currently available therapeutic options.

The timing, dynamics and molecular basis of the metastatic process have been representing matter of interest and intense investigation for ages. In breast cancer, clinical studies have provided evidence that the presence of single disseminated cancer cells in bone marrow or lymph nodes impacts prognosis significantly [13]. With the advent of first generation techniques for single cell analysis, genetic profiling revealed that tumor cells disseminated to bone marrow and detected before and after the manifestation of metastasis might have started to diffuse during tumor initiation and continued to evolve in parallel with the primary tumor [14,15,16]. By performing comparative genomic analysis of primary and metastatic lesions, it has been estimated that the dissemination of cancer cells from primary to distant sites occurs 2–4 years before diagnosis of the primary tumor [17]. Studies in transgenic mouse models [18] and in patients with in situ carcinoma of the breast [19] firmly corroborated the concept that hematogenous dissemination is an early event in breast cancer progression, and unveiled the mechanism at the basis of early diffusion, which seems to involve cell density, progesterone receptor signaling and HER2 signaling, irrespective of the breast cancer subtype [20]. Following such groundbreaking findings, it has becoming increasingly clear that metastasis-initiating breast cancer cells do not necessarily arise from an advanced variant clone pre-existing in the primary tumor, sparkling interest in the molecular signs that distinguish the metastatic rather than proliferative events of breast cancer. However, the possibility to systematically study the precursor cells of metastasis has been hampered for many years by their extreme rarity and the lack of specific markers to distinguish them within the tumor bulk.

Nowadays, technological development has made it possible to detect cancer cells originating from solid neoplasms in specimens of reservoir tissues and circulating fluids, such as bone marrow, lymph nodes or blood. Research in this field has been focusing on rare disseminated tumor cell detection and enumeration for years, in an attempt to find a gold standard method and to develop cell-based classifiers for supporting clinical decision during patients follow-up. Limitations still exist mainly due to technical and biological variability, and the choice of one of the other method may depend on the experimental question or clinical aim. In particular, the analysis of tumor cells disseminating through the bloodstream, i.e., circulating tumor cells (CTCs), may offer a minimally invasive and repeatable approach for improving risk stratification and evaluating prognosis or response to treatment, for three fundamental reasons: (1) they can spread early from a tumor mass, (2) are actively shed during tumor progression, and (3) might contribute to disease relapse after local and systemic adjuvant therapies. CTCs embody the ideal biomarker as they represent a less invasive substitute for conventional tissue biopsy, can be propagated to give rise to novel cancer models for basic and preclinical studies, and analyzed in blood samples by using cytometry, genomics, transcriptomics and proteomics-based techniques. Thus, CTC analysis can give insight into cancer evolution, support the personalization of current therapies and foster the design of novel CTC-targeting compounds. In breast cancer, the molecular analysis of CTCs might complement and possibly improve the information provided by the study of the tumor mass. For its biological and clinical diversity, breast cancer has actually represented the best example of successful application of molecular testing, mainly based on the gene expression profiling of the primary tumor, in terms of disease subtyping, identification of tumors susceptible to targeted treatments, and prognostication. Considering that excellent points have been made in this field in recent years, but the application of gene signatures in the clinical context is not broadly diffused, yet, what we can expect from CTC transcriptome analysis and comparison between the “solid” and “liquid” phase of tumor progression in breast cancer?

The aim of this review is to offer the reader an overview of the opportunities and challenges arising from CTC transcriptome analysis in breast cancer. Current use of multigene-based tests derived from microarray studies, data availability on CTC gene expression profile with preceding and next-generation sequencing techniques, and the significance of CTC-related signatures in cancer progression and in metastasis biology, are the topics covered in this work. Technical issues related to CTC detection and transcriptomics are also discussed with a view to potential applications and clinical use.

## 2. Breast Cancer Gene Signatures

Breast cancer encompasses a heterogeneous group of diseases that differ in a series of tumor-intrinsic features, including histological, immunohistochemical and molecular profile, and in tumor-extrinsic features, such as microenvironmental and systemic factors [21]. Breast cancer heterogeneity largely affects prognosis and response to specific treatments, with subsequent implications for patient management and probability of survival.

More than two decades ago, the traditional histology-based classification of breast cancers has been refined and complemented with the definition of molecular classes. The combination of histopathological parameters, expression patterns of hormone receptors (Oestrogen and/or Progesterone Receptors; ER/PR) and Epidermal Growth Factor Receptor 2 (HER2/*neu*), and genomic and transcriptomic profile data brought to light the existence of several breast cancer subtypes. Seminal works have described molecular subtypes defined by transcriptional signatures that partially recapitulate the original immunopathological classes, while adding a further level of detail. As a result of two seminal gene expression profile studies, five breast cancer intrinsic subtypes were identified by the hierarchical clustering of genes characterized by significantly greater variation in expression among different breast tumors than between paired pre- and post-chemotherapy tumor samples: luminal A (ER+/PR+, low Ki67 index, histological grade I/II), luminal B (ER+/PR+, HER2 expression variable, high Ki67 index, histological grade II/III), HER2-enriched (ER−/PR−, HER2+, histological grade II/III), basal-like (ER−/PR−/HER2−, histological grade III), and normal breast-like (ER+/PR+, HER2−, histological grades I–III, low Ki67 index) [22,23]. The two luminal subtypes (A and B) mainly encompass ER+ cases and are distinguished by the presence of genes regulated by the ER signaling pathway typical of the luminal epithelial layer of the mammary gland. The luminal A compared to the luminal B subtype is associated with higher levels of *ESR1*, ER, and ER-regulated genes, decreased proliferation, and better overall outcome [22,23]. Other studies have suggested that the distribution of luminal tumors may be modeled as a continuum along which ER-regulated elements and proliferation are inversely related rather than grouped in two distinct categories [24,25]. The HER2-enriched subtype partially overlaps with HER2+ tumors as defined by immunohistochemistry [22] and is characterized by high expression of HER2 oncogene and adjacent loci in the 17q12-q21 amplified genomic sequence. The basal-like subtype is defined by the expression of genes typical of the outer or basal epithelial layer of the mammary gland, such as cytokeratins 5/6 and 17 and the Epidermal Growth Factor Receptor EGFR, and is associated with the shortest relapse-free survival [26]. Although the basal-like and the triple-negative (ER−/PR−/HER2−) cohort share similarities, molecular disparities between the two types of classification and intra-class heterogeneity have been evidenced [27,28]. Cluster analysis of gene expression profiles from 21 breast cancer data sets, corresponding to 587 triple-negative breast tumors, identified six groups exhibiting preferential responses to specific chemotherapeutic regimens, as well as differential expression of basal-specific, immunomodulatory, mesenchymal, mesenchymal stem-like, and androgen receptor-related genes [29]. Finally, the normal-like molecular subtype seems to resemble the normal epithelial tissue and may comprise cases in which samples contain large amounts of non-tumor tissue [22]. Another breast cancer subtype called claudin-low emerged from an integrated analysis of human and murine mammary tumors a few years after the establishment of the first molecular classification [30]. Claudin-low tumors are characterized by the low to absent expression of luminal differentiation markers, high enrichment for epithelial-to-mesenchymal transition (EMT) markers and immune response genes, cancer stem cell-like features, and marked immune and stromal cell infiltration [31,32,33]. According to the initial classification, claudin-low was interpreted as a sixth subtype, analogous to the intrinsic subtypes previously identified. However, a recent study showed that when stratified by intrinsic subtype, claudin-low tumors display characteristics associated with their own intrinsic subtype, thus implying that tumors can carry a claudin-low phenotype in addition to their intrinsic subtype, and suggesting that individual tumors are not simply claudin-low or non-claudin-low, but may show claudin-like features with varying degrees [34].

Breast cancer subtypes are associated with distinct patterns of metastatic spread. Multivariate analysis on 3726 early stage breast cancers highlighted that bone is the most common metastatic site in all subtypes except basal-like tumors, that luminal/HER2 and HER2-enriched tumors are associated with a significantly higher rate of brain, liver, and lung metastases, that basal-like tumors have a higher rate of brain, lung, and distant nodal metastases, but a significantly lower rate of liver and bone metastases, and that triple-negative non-basal tumors demonstrate a similar pattern, but are not associated with fewer liver metastases [35].

Molecular classification has extremely influenced the way in which breast cancer is currently regarded by clinicians and research scientists. In 2002, microarray gene expression analysis was performed on 117 breast tumors with no evidence of tumor cells in local lymph nodes at diagnosis with the aim to improve the identification of patients at risk of metastatic recurrence and the classification of those who could better benefit or be spared from adjuvant therapy. The authors identified a 70-gene signature consisting of genes regulating cell-cycle progression, invasion, metastasis and angiogenesis, such as those coding cyclin E2, MCM6, metalloproteinases MMP9 and MP1, RAB6B, PK428, ESM1, and the VEGF receptor FLT1, all significantly up-regulated in patients with poor prognosis, i.e., with the appearance of distant metastasis within 5 years [36]. Importantly, the 70-gene signature was able to provide prognostic information beyond standard clinical assessment. Moreover, a risk model that incorporates the gene expression-based “intrinsic” subtypes luminal A, luminal B, HER2-enriched, and basal-like was developed in order to improve standards for breast cancer prognosis and prediction of chemotherapy benefit [37,38]. In 2017, the American Joint Committee on Cancer (AJCC) recognized the need to incorporate gene expression prognostic panels into the TNM staging system (eighth edition). 

As a result of the body of knowledge provided by such studies, molecular predictors were developed and entered the clinical practice. The Oncotype DX^®^ Recurrence Score (RS) [39], MammaPrint^®^ [40], EndoPredict (EP/EPclin) [41], Prosigna^®^ Risk of Recurrence Score [37], and Breast Cancer IndexSM (BCI) are now the five commercially available multigene assays endorsed by clinical practice guidelines able to provide low-risk scores for the management of luminal early breast cancer. Risk scores can be used regardless of the tumor size to downstage hormone receptor-positive, HER2 negative and lymph node-negative primary breast cancers, placing them into the same prognostic category as T1a-T1b N0 M0 carcinomas [42]. Currently, Oncotype DX^®^ (21 genes) and MammaPrint^®^ (70 genes) help clinicians to make decision on the kind of treatment in two specific clinical settings: adjuvant chemotherapy in patients with early ER+/HER2− tumor and adjuvant extended hormone therapy in post-menopausal patients with ER+ tumor, thus helping to de-escalate systemic therapy. Multigene-panel tests have been incorporated into clinical practice to complement traditional pathology and guide clinical decisions, but limitations to the broad diffusion of the 70-gene and the 21-gene signatures unfortunately still exist, and they include costs, need for adequate tissue sampling, the requirement to send samples to a reference center and, in the case of Oncotype DX^®^, the existence of an intermediate group for which the value of adjuvant chemotherapy is not clear. Moreover, such gene signatures work for ductal carcinoma while alternative panels of markers seem to better classify and mirror the clinical outcome of lobular carcinoma [43,44]. 

The status of HER2 proto-oncogene in breast cancer is assessed to select patients eligible for targeted therapy with anti-HER2 monoclonal antibodies. Signatures derived from HER2-positive cohorts would be useful to predict responses to the anti-HER2 humanized monoclonal antibody trastuzumab [45,46], for which cardiotoxicity and limited efficacy in preventing metastases has been reported. According to the American Society of Clinical Oncology (ASCO) and College of American Pathologists (CAP), the HER2 test positivity is defined by protein overexpression (score 3+) at immunohistochemistry (IHC) and/or gene amplification at in situ hybridization (ISH). In 15–20% of cases, breast cancers show the overexpression of HER2, usually due to gene amplification [47]. On the contrary, tumors with IHC scores 0 and 1+, or 2+ with a negative ISH, are clinically HER2-negative [48]. Although this dichotomization drives the decision of whether or not to administer trastuzumab, it was observed that HER2-negative breast cancer is characterized by a wide spectrum of HER2 expression levels and that advanced breast cancer patients harboring HER2-low expression can benefit from the administration of novel anti-HER2 antibody–drug conjugates [49,50]. As discordance in HER2 status between primary and recurrent breast cancer has been documented [51,52,53], and molecular heterogeneity associated with differential survival time after treatment with trastuzumab exists within the HER2+ population [54,55], retesting metastatic breast cancer for HER2 status, even in the HER2-low category, and molecular characterization of residual disease are gaining increasing attention [56,57].

Triple-negative breast cancer has limited treatment options and progresses rapidly. Defining sensitivity in terms of complete response has been an elusive goal, and signatures able to identify patients with favorable prognosis have not been validated yet. One possible explanation is that there may be multiple mechanisms of drug resistance and one single signature is not able to identify all of them. Despite their unfavorable prognosis when regarded as a single group, many triple-negative breast cancers are instead highly sensitive to chemotherapy, and in the neoadjuvant setting they have an increased response rate compared with other breast cancer subtypes, although their survival is still worse, a phenomenon commonly referred to as the triple-negative paradox [58,59]. However, gene profiling in triple-negative breast cancer is unlikely to find patients who do not need adjuvant chemotherapy and sub-classifications have not yielded advantages in treatment decision so far, although an increasing number of studies attempting to decipher breast cancer heterogeneity have enabled to appreciate the biology of this aggressive subtype and have started to highlight a more promising therapeutic scenario compared to standard chemotherapies for the identification of subgroups of patients to be treated with new specific therapies [60,61].

With a view to the biological significance of breast cancer gene signatures, it should be considered that the primary determinants of all the signatures are proliferation, ER-status, HER2-status, and, less prominently, angiogenesis, invasiveness, and apoptosis. The vast majority of these have been built using supervised classification systems in which gene expression data are paired with survival information. The fact that clinically relevant breast cancer signatures significantly correlate with disease course although they do not share the same list of genes suggests that these signatures detect the same biological processes and pathways involved in metastasis. However, it is important to note that gene expression profiles derived from the tumor bulk reflect an average value of the expression level for each gene that is dependent on the number, location and type of cells surveyed. Therefore, the specific nature of a tumor may be better defined by its location within a multidimensional continuum, in which the canonical subtype-defining signatures represent vertices. In 2012, Curtis and colleagues published the genomic and transcriptomic profiles of 2000 human breast cancer samples by using an integrated analysis of copy number variations and gene expression patterns, describing additional subtypes of breast cancer derived from the impact of somatic copy number aberrations on the transcriptome [62]. Ten integrative clusters, named IntClust 1–10, emerged by means of copy number aberration profile and transcriptomic landscape analysis, enabling the refinement of the distinction among subgroups of patients with different clinical outcomes. An ER+ subgroup with specific alterations was found to exhibit a high mortality risk, whereas ERBB2-amplified cancers including both HER2-enriched (ER−) cases and other luminal (ER+) cases formed a cluster that improved the definition of the ERBB2 intrinsic subtype by grouping additional patients expected to benefit from targeted therapy. Groups with low genomic instability and better prognosis or with intermediate prognosis emerged from the remaining case series, including the majority of basal-like tumors, mostly with high genomic instability and relatively good long-term outcomes (after 5 years). It is clear that multiple levels of breast cancer heterogeneity exist and are worth being investigated to unveil all possible clinical consequences and opportunities. The polyclonal and evolving nature of breast cancer should be taken into consideration during clinical decision-making. Thus far, progress in the treatment of metastatic breast cancer has mainly occurred as a result of clinical trials in which patients are grouped by classical pathological subtyping. Perhaps most pressing is the need to understand the biological similarities and differences between primary tumors and their metastatic descendants. Thus, an accurate comparison of the molecular hallmarks that govern primary tumor growth with those that govern the dissemination and outgrowth of metastases through CTC and metastasis mining approaches is essential to enable the development of therapies specifically designed to prevent or treat metastatic disease.

## 3. Circulating Tumor Cells: The Kinetic Phase of Metastasis

CTCs are key players of an intermediate step of tumor progression that is crucial to the establishment of metastasis, as it starts with the intravasation of a subset of cancer cells able to leave the tumor site and spread through the bloodstream. For this reason, CTCs have been postulated to be enriched for metastasis-initiating cells and are regarded as the direct precursors of metastasis [63]. In breast cancer, a subset of CTCs with a CD44+CD47+Met+/− expression pattern was found to form multi-organ metastases after injection into the femurs of immunocompromised mice [64]. In another work, a population of CTCs, selected for a panel of markers consisting of Her2+/EGFR+/HPSE+/Notch1+ and propagated in vitro, showed a propensity to metastasize to the brain compared with the parental counterpart when injected in the circulatory systems of mice [65]. The results of such studies have clearly indicated that CTCs do not have all the same metastatic potential and organotropism. However, CTC subsets were selected on the basis of specific protein expression patterns and data on CTC metastagenicity at the single cell level are still lacking.

**Figure 1:**
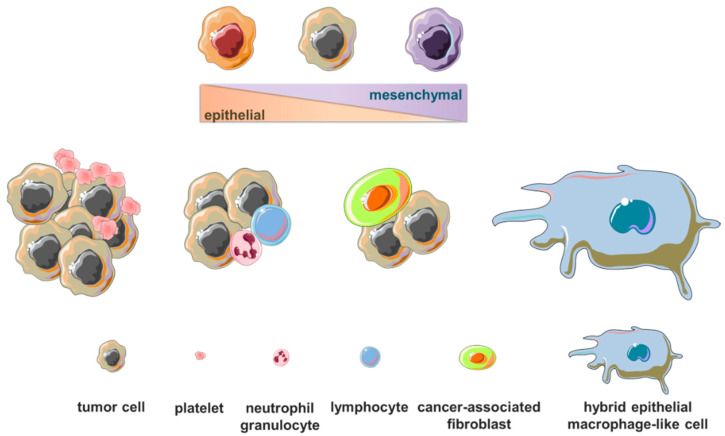
Breast cancer cells circulating in blood are heterogeneous. Circulating tumor cells (CTCs) may undergo epithelial-to-mesenchymal transition and revert their phenotype, also entering several intermediate states. Tumor cells can disseminate either separately from each other, as single CTCs, or forming homotypic and heterotypic clusters; CTC clusters are made by aggregates of tumor cells only, sometimes surrounded by platelets, and by aggregates of tumor cells and other cell types, such as neutrophil granulocytes, cells expressing the common leukocyte antigen CD45, as in lymphocytes, and cells expressing markers of cancer-associated fibroblasts. Cells with larger size than tumor cells expressing both epithelial and monocyte/macrophage markers are associated to the presence of CTCs, and they are called hybrid epithelial/tumor macrophage-like cells. (Cartoon depicting cell types was adapted from templates available in smart.servier.com, accessed on 26 October 2022).

Metastasis is an intricate and inefficient process whose success is dependent on the ability of cancer cells to orchestrate a series of molecular mechanisms, overcome anatomical obstacles and fruitfully interact with different microenvironmental elements [66,67,68,69]. In an attempt to explain the stochastic behavior that seems to characterize metastasis, the fate of disseminating tumor cells has been intensively investigated in studies based on cancer models, which highlighted mechanical forces, cell fitness in the circulatory system and post-extravasation events as main rate-limiting steps of cancer cell diffusion [70,71,72,73,74,75,76,77,78]. In a breast cancer rat model, it was estimated that primary mammary adenocarcinomas shed some millions of cells per gram of tissue every 24 h [79]. Decades later, the circulatory clearance of single CTCs and CTC clusters obtained from a human lung-tropic breast cancer cell line was measured in ad hoc experiments by in vivo flow cytometry upon injection of the two CTC subpopulations in the tail vein of mice, showing that the overall half-life in blood vessels ranged from 6 to 30 min [80]. Consistently, in CTC kinetics experiments, another study reported that CTC count greatly drops 1 h after injection of breast cancer cells in the tail vein, while metastasis formation in the lungs still occurs [81], thus indicating a rapid CTC clearance. Moreover, the frequency of clusters of CTCs and white blood cells in mouse models was found to be higher when drawing blood from a tumor draining vessel as opposed to sampling from downstream locations, suggesting that CTC clusters may temporarily remain entrapped in the capillary bed before reaching peripheral tissues [82]. Hemodynamic forces are also implicated in the success of metastasizing cells. Experiments in zebrafish embryo models allowed demonstrating that blood flow tunes both the arrest and extravasation of CTCs and the endothelium remodeling around arrested tumor cells [83]. Findings originating from such studies are fundamental starting points to understand the kinetics of hematogenous dissemination. However, it should be considered that the majority of them have in common an experimental approach for metastasis modeling based on tumor cell injection in the vasculature, which obviously does not allow for fully recapitulating the multiple steps of the metastatic process and to appreciate possible molecular features acquired before or during the intravasation phase, as is the case in spontaneous metastasis experiments. An original approach was recently developed to measure the kinetics of endogenous CTCs during continuous exchange between tumor bearing and tumor-free mice through a peristaltic pump. By tracking CTC transfer rates, the authors extrapolated half-life times in the circulation ranging from 40 to 260 s and intravasation rates between 60 and 107,000 CTCs/hour in several mouse models [84]. Another recent work showed that, compared to intravascularly injected tumor cells, disseminating tumor cells developed spontaneously are retained longer and at higher frequency, extravasate from the lung vasculature more quickly and have a greater chance of survival after extravasation [85], corroborating the involvement of other microenvironmental factors in dissemination, such as primary tumor-associated macrophages [85,86]. The importance of cancer cell–cell cooperation and tumor-associated microenvironment cells in CTC survival has been largely documented [87], firmly demonstrating that the formation of homotypic and heterotypic clusters in experimental models endows cancer cells with higher colonization ability and proliferation rate than single CTCs. A cartoon depicting CTC subtypes is available in Figure 1.

In the clinical setting, estimated CTC half-life seems to be consistently brief as those observed in experimental models. Cytokeratin-positive CD45-negative CTCs were counted in 40 to 60 mL of blood samples collected shortly before or immediately after removal of the primary tumor and at subsequent intervals from a small group of patients with primary breast carcinoma. By combining two analytical models, CTC half-life was estimated to range from 1 to 2.4 h [88]. Another group, by working on a mathematical model to measure the contribution of a panel of parameters to the success rate of the metastatic cascade, found that the most critical parameter governing the formation of clinical metastases in breast cancer is the survival duration of CTCs [89]. 

Measuring CTC intravasation rate and half-life time in the circulatory system is important to understand the timing of CTC seeding in distant organs. It is commonly assumed that the statistics of CTC detection follows a Poisson distribution [90,91,92]. This implies that CTCs are homogeneously mixed with blood components and that their average number in circulation does not change significantly over time. Actually, preliminary data have been provided in the last two years in support of a dissemination kinetics model characterized by states of relatively high to low rate of CTC release in blood rather than as a steady Poisson distribution. By using in vivo flowcytometry, it was shown in preclinical animal models of breast cancer and melanoma that the CTC number changes during short-term measurements [93], and similar fluctuations were observed in an orthotopic xenograft model of prostate cancer, where CTC load was higher during the early stage of tumor progression and underwent daily oscillations [94], and in multiple myeloma and Lewis lung carcinoma xenograft models [95], highlighting the importance of repeated sampling to obtain reliable CTC quantification. A recent paper shed light on the effect of circadian rhythm in determining the frequency of tumor cell intravasation in both patients with early or advanced breast cancer and mouse models injected with highly metastatic breast cancer cells, showing that hematogenous dissemination mainly occurs during sleep and that CTCs with colonization ability are those generated in the rest phase and proliferate actively [96]. 

The diffusion of tumor cells from the primary tumor to the circulatory system is definitely a complex process influenced by physical and biological factors. A broad understanding of the molecular mechanisms at the basis of cell-cell and cell-matrix interactions, intravasation, survival in blood and extravasation, requires considerable effort and the application of multidisciplinary approaches. CTC half-life and timing of blood-borne dissemination turned out to be crucial factors in the metastatic process, which may affect CTC recovery after blood sampling, the accuracy and completeness of information on the disease status that can be retrieved through CTC analysis, and the efficacy of therapies. A huge amount of experimental data is needed to unveil the mechanisms of hematogenous dissemination, and the novel body of findings should be linked to clinical observations in order to provide evidence-based guidelines for the standardization of pre-analytical workflows in CTC analysis and the development of CTC profile-based effective therapies to stop cancer cell dissemination. At present, still little is known about the transcriptome profiles that drive hematogenous dissemination and therapy resistance in disseminating cells.

## 4. CTC Detection and Clinical Significance of CTC Count in Breast Cancer

CTCs appear to be the most underrepresented population of cells that can be detected in the blood of patients affected by a solid tumor. Although their presence was first documented approximately 150 years ago thanks to the observations reported by the pathologist Thomas Ashworth [97], the CTC topic was not widespread in cancer research and CTC analysis was not introduced in oncological trials until recently. By using flow cytometry or reverse transcription PCR, the CTC frequency in peripheral blood samples of patients with breast cancer was estimated in some initial studies as low as 1 cell per 10^5^–10^7^ leukocytes [98,99,100,101]. Due to CTC rarity and the initial lack of sensitive technologies, it was only in the last two decades that some cytometric and molecular techniques have found large diffusion in CTC studies, and they are still subject of technological efforts aimed at improving accuracy in detecting rare disseminating cells in body fluids and tissue. Both biological and physical features lie at the basis of techniques to separate CTCs from other cell types. First, breast and other carcinomas are of ectoderm origin and differ from leukocytes, which have mesodermal origin, in their transcriptome profile, and, therefore, in the pattern of tissue-specific proteins expressed on the cell surface or at intracellular level. Therefore, immunoaffinity assays for CTC detection are mainly based either on cell labeling with magnetic particles functionalized with one or more antibodies against a specific surface marker, such as EpCAM, HER2 and EGFR, or on leukocyte depletion using the same cell selection principle based on cell labeling with antibodies against CD45 and other cluster of differentiation antigens. Physical parameters have also been applied considering that CTCs have larger size than the majority of white blood cell populations, whose diameter in isotonic solution is less than 10 μm [102]. A recent paper demonstrated that CTC diameter is significantly different among several cancer types and that EpCAM-selected cytokeratin-positive CTCs in women with breast cancer have smaller median diameter (12.4 μm) compared to tumor cells detected in the liquor (13.4 μm) and to in vitro cultured cell lines (18.4 μm) [103].

The enrichment step is an essential prerequisite to proceed with CTC detection. However, each method can be affected by a selection bias when based on the biological and physical properties of tumor cells and normal blood cells. Alternative technologies have recently been developed to enable the transfer of virtually the entire nucleated cell fraction from blood to microscope slides, reducing the impact of multistage processes that may lead to cell damage or loss [104,105,106]. However, a second analytical step is needed in any case to distinguish CTCs from non-target cells, and at this stage the choice of the panel of markers, either at protein or gene expression or DNA level, is crucial for the biological sensitivity of the assay since breast cancer is highly heterogeneous. Basically, CTCs are detectable by (i) in situ techniques, such as cytological staining to recognize atypical cells by cyto-morphological analysis, as also immunocytochemistry and immunofluorescence protocols to assess the expression of a panel of protein markers, and by using interphase FISH or RNA hybridization to detect chromosome rearrangements or specific fusion transcripts or gene transcripts, and by (ii) low-density or high-density gene expression arrays, PCR-based detection of specific mutations, and RNA or DNA sequencing, which, differently from in situ techniques, require cell lysis and nucleic acid purification or amplification, either at the single cell level, if the cell separation technology enables the retrieval of pure single cells, or as a pool of CTC-enriched cells, i.e., including leukocytes and other non-target cells that clearly influence the assay specificity.

Advancements in technology are now increasing the possibility to perform single cell analysis on CTCs in breast cancer. However, at present, we can benefit of a body of knowledge resulting from studies mainly based on CTC enumeration, and data on the clinical significance of CTC gene expression profile in breast cancer are still scarce. Importantly, accumulating evidence from clinical studies has firmly demonstrated that CTCs are an independent prognostic factor in metastatic breast cancer. By identifying a threshold of 5 CTCs detected in 7.5 mL of peripheral venous blood by EpCAM-based immunomagnetic capture followed by immunostaining for cytokeratins (CellSearch^®^ System), it was shown that CTC count at baseline and increased levels of CTCs at any time point during treatment are strongly associated with higher risk of disease progression and cancer-related death compared to patients with less than 5 CTCs [107,108]. A subsequent pooled analysis involving 20 studies confirmed the clinical validity of CTCs in metastatic breast cancer and showed that CTC count also improves prognostication when added to clinico-pathological predictive models [109]. More recently, a study involving 2436 patients classified those with at least five CTCs as stage IV-aggressive due to significantly inferior overall survival compared to those with less than five CTCs who were defined as stage IV-indolent [110], fostering further studies aimed at refining CTC-based risk stratification by monitoring changes in CTC levels across time points over the course of therapy [111]. Results from ongoing clinical trials indicate that CTC count may be a reliable biomarker for guiding the choice between chemotherapy and endocrine therapy as the first-line treatment in hormone receptor-positive HER2-negative metastatic breast cancer [112], but further investigation is still needed to confirm the utility of CTCs as a pharmacodynamic biomarker of treatment efficacy [113]. In patients with operable or locally advanced breast cancer, CTC positivity is reported in 5–29% of cases when using EpCAM-based enrichment or direct immunostaining of cytological blood samples to detect epithelial markers [114,115,116,117,118]. Similarly, in the early stage clinical setting, the presence of CTCs predicts early recurrence and decreased overall survival [117,119]. Moreover, a pooled analysis of individual data from 3173 patients revealed that patients with CTCs (20%) had more aggressive tumors compared to those with CTC-negative samples, and confirmed that the presence of CTCs was an independent predictor of poor disease-free, overall, breast cancer-specific, and distant disease-free survival [120]. In patients under treatment with neoadjuvant chemotherapy for large or locally advanced breast cancer, CTC detection before administration of chemotherapy again was found to represent an independent prognostic factor for reduced metastasis-free and overall survival [115]. In patients treated with adjuvant chemotherapy, increase in CTC count is still confirmed as a predictor of relapse [121]. CTCs were also found in the peripheral blood of women without current evidence of disease following surgical eradication [88], but the clinical utility of CTC enumeration in early breast cancer and in the absence of disease has not been determined yet. To this aim, it is important to consider that the percentage of CTC-positive cases in non-metastatic breast cancer may reach 90% when filtering large volumes of blood through leukapheresis followed by immunostaining for cytokeratins (90%) [122], 88% when coupling EpCAM-based Immunomagnetic Enrichment with Fluorescence-Activated Cell Sorting (IE/FACS) [123], 78% when size-selecting cells by filtration of blood samples through porous membranes [124], or 75% when screening cytological samples of peripheral blood mononuclear cells with epithelial and breast cancer-associated protein and genetic markers [106].

In breast cancer, CTC detection and count are useful tools for tumor staging, as prognostic markers, for treatment monitoring and for post-treatment surveillance. Technical approaches and tests for CTC enumeration have been developed based on large cohort studies and entered the clinical routine in some research hospitals and diagnostic laboratories. The CellSearch^®^ system was approved by the United States Food and Drug Administration for aiding the monitoring of patients with metastatic breast cancer. Other immunoaffinity-based technologies that found research application are IsoFlux™ Rare Cell Access System, which combines multiple antibody-based magnetic capture kits with flow-cytometry, and GILUPI CellCollector^®^, a medical wire designed to capture CTCs in vivo while being inserted in the cubital vein for 30 min. Technologies developed to exploit tumor cell physical properties and that are finding large use in translational research laboratories are ISET^®^, ScreenCell^®^, CellSieve^TM^, which utilize a filtration-based size exclusion approach, and Parsortix^®^ and DEPArray^TM^, microfluidics devices for cell separation based on size or dielectrophoretic activity, to mention a few. Determining the transcriptional profile of CTCs represents now the next step toward their comprehensive utilization in the clinical setting, as the interrogation of their molecular profile is expected to increase the number of clinically relevant information while overcoming tumor heterogeneity-related biases associated with tissue sampling and bulk analysis for biomarker assessment.

## 5. CTC Gene Expression Profile Studies in Breast Cancer

First reports on the detection of tumor cell-related transcripts in blood samples had shown that cell-free mRNA molecules coding for cytokeratin could be quantified in the peripheral blood mononuclear cell fraction of women with a diagnosis of breast cancer. Research scientists have started to couple cytometric and immunocytochemistry techniques for CTC enrichment to protocols for nucleic acid analysis. The next paragraphs show the results of gene expression profile studies intended not only for CTC detection, but also for CTC characterization and the development of CTC-related signatures.

### 5.1. Experimental Strategies to Identify CTC Detection-Specific Genes in Breast Cancer

CTC detection through gene expression profile analysis requires some strategies to distinguish CTC-specific transcripts from background signals. In 2005, a research group performed the first microarray experiment on three patients with a metastatic colorectal, prostate, and breast cancer, presenting with at least 100 CTCs in 7.5 mL of their blood [125]. The patient with breast cancer, in particular, was positive for hormone receptors and had 3700 CTCs as assessed by immunostaining for cytokeratins upon EpCAM-based immunomagnetic capture. By comparing RNA samples of the CTC-enriched blood fraction with RNA extracted from residual blood obtained after completing the CTC depletion step, the authors first generated a global gene expression profile and then identified a list of cancer-specific and/or CTC-specific genes. Regarding the patient with breast cancer, 71 genes were expressed in the CTC-enriched sample and were undetected in the matched CTC-depleted fraction. The two members of the EpCAM family (*TACSTD1* and *TACSTD2*), the surface marker used to select for CTCs, and keratin 19 (*KRT19*), which is frequently used to identify CTCs of epithelial origin, were among the upregulated genes. Moreover, mammaglobin 1 (*MGB1*/*SCGB2A2*) was among the genes specifically expressed in the metastatic breast cancer patient and absent in the other two clinical cases, although the matched CTC-depleted blood sample was also called as positive. Then, samples enriched for CTCs from 74 metastatic cancer patients, 13 of them with breast cancer, and 50 normal donors, were used to confirm by reverse transcription-quantitative PCR (RT-qPCR) the expression of those genes selected as CTC-specific based on the previous global analysis. In breast cancer patients, the median and mean CTC numbers in 7.5 mL were 4 and 104, respectively, while donors were assumed to have no CTCs. Genes with no significant expression in the majority of the normal donors and exhibiting expression patterns associated with a particular cancer type were 16, among them *AGR2*, *S100A14*, *S100A16*, and *FABP1* identified as those with the highest discrimination power between cases with cancers and donors. *SCGB2A1*, *SCGB2A2*, and *PIP* genes showed association with breast cancer, in addition to *S100A14*, *S100A16*, and *CEACAM5*, which were associated with colorectal cancer as well. The authors also observed that the accuracy in classifying the tissue of origin among the three cancer types was 79.3%. Ten years later, the technical validity of a pipeline [126] based on the immunomagnetic-mediated isolation of EpCAM/MUC-1 positive CTCs followed by the chemical depletion of the majority of leukocytes, i.e., cells non-specifically bound to the magnetic particles, and by cDNA hybridization on a sensitive microarray platform that was suitable for degraded or low-input RNA samples, was demonstrated. The technical reliability of the protocol was first assessed in breast cancer cell lines by comparing cells collected from a cell culture with those spiked-in and recovered by immunomagnetic selection from blood samples of healthy donors. Samples from seven patients with metastatic breast cancer were then analyzed with the same pipeline and in parallel with the CellSearch^®^ or the AdnaTest^TM^ kits. The number of CTCs in 5 mL of blood ranged from 0 to 200 in six patients, while the only case analyzed by AdnaTest^TM^ was called as negative according to the expression of a panel of 7 epithelial-, breast cancer- and EMT-related genes. Unsupervised hierarchical clustering analysis of gene expression profiles using genes from the PAM50 panel [37] showed that there was no correlation between the number of CTCs estimated by CellSearch^®^ and the expression levels of PAM50 genes. Interestingly, all seven cases expressed high levels of a cluster of genes from PAM50, including *SLC396*, *MYC*, *MDM2*, *BAG1*, *CXXC5*, *BCL2*, *ORC6L*, *PTTG1*, *GPR160*, *PHGDH*, *NAT1*, and *BLVRA*.

Two works in 2010 reported the results of a comparative gene expression analysis between blood samples of healthy donors and of patients with stage I to IV breast cancer. In one study [127], microarray analysis was first performed to identify a panel of differentially expressed genes between breast and ovarian cancer cell lines and the PBMC fraction of healthy donors. A validation RT-qPCR test on a subpanel of genes was subsequently run to characterize PBMCs of patients with stage I to III breast cancer, purified through the OncoQuick^®^ kit, a combination of density gradient centrifugation with size-based filtration, from blood samples collected before starting primary systemic therapy and at disease relapse. The authors considered those genes over-expressed in more than 10% of the patients with recurrent breast cancer and identified a panel of six genes for CTC detection: *CCNE2*, *DKFZp762E1312*, *EMP2*, *MAL2*, *PPIC*, and *SLC6A8*. The six-gene panel was able to detect 81% of the breast cancer patients with recurrence and 29% of those at initial diagnosis, all cases positive for at least one gene. In the other study published in the same year [128], the approach for CTC detection was based on a functional test called collagen adhesion matrix (CAM) assay, which enables us to select cells with invasive properties based on their ability to remove and ingest CAM fragments. CTC count per milliliter of blood was 0 in healthy donors, and CTC count and positivity frequencies ranged from 8 to 119 and 27% to 87% in patients with breast cancer at stage I to III. In samples with at least 60 CTCs/mL, *KRT8*, *KRT16*, *KRT1*, *KRT19*, *TERT*, *MUC16* (*M17S2*/*CA125*), *CD44*, *TWIST1*, *TACSTD1* (*EPCAM*/*CD326*/*ESA*/*HEA125*/*GA733*), *DPP4* (*CD26*), *ESR1* and *PGR* genes were upregulated compared to healthy donors. Such genes are mainly related to epithelial and breast tissues and to the EMT process. A novel panel of genes for CTC detection, alternative to epithelial, breast tissue, and EMT process-related markers, was identified by comparing RNA sequencing data of IE/FACS isolated CTCs from patients with stage II-III breast cancer with those of matched primary tumor specimens and blood samples from healthy donors: CTC samples clustered together and separately from primary tumor and healthy donor samples, and *HBB*, *HAND2*, *OR52H1*, *CATSPER4*, and *CLRN1* emerged as the top five most significantly upregulated genes in CTCs compared to primary tumors [123].

Gene expression profile data of normal breast, breast cancer and CTCs were also interrogated by another research group to derive a diagnostic signature [129]. The authors focused on genes strongly expressed in breast cancer-derived tissues and virtually not expressed in healthy donors, and by interrogating RNA-sequencing and microarray gene expression data sets they found a 17-gene signature, including breast lineage-specific transcripts (*PGR*, *SCGB2A1*, and *PIP*) and transcripts highly expressed in breast cancer (*MGP* and *EFHD1*), as well as gene products implicated in endocrine signaling (*SERPINA3* and *WFDC2*), endocrine drug resistance (*AGR2*), cancer growth and metastasis (*MUC16* and *TMPRSS4*), cellular signaling (*FAT1*, *FAT2*, *SFRP1*, and *SFRP2*), epithelial-derived cytokines (*CXCL13* and *CXCL14*), and oncofetal antigens (*PRAME*) [129]. Single-cell RNA-sequencing revealed that the expression of the 17 markers was heterogeneous among 15 individual CTCs isolated from blood samples of 10 women with metastatic breast cancer and negligible when assessed in five single WBCs. The signature was then validated in female healthy donors and in patients with stage I to IV breast cancer upon microfluidics-based enrichment and EpCAM-dependent capture of CTCs by using the herringbone CTC-iChip [130], and after optimizing digital droplet PCR detection signals by subtracting background signals detected in a preliminary cohort of 30 female healthy donors. At a specificity of 100%, the CTC gene expression-based test showed step-wise increase in sensitivity from 19% in stage I to 67% in stage IV cases.

In another study [131], microfluidics-based multiplex qPCR array of 64 cancer-related genes was performed on RNA isolated from EpCAM+/CD45− cells and matched leukocytes, defined as EpCAM−/CD45+ cells, collected from blood samples of patients with metastatic breast cancer through immunomagnetic EpCAM-dependent enrichment followed by FACS. CTC clustered separately from leukocytes and the series of genes *CCND1*, *EPCAM*, *MUC1*, *TFF3*, *AGR2*, *ERBB2*, *TFF1*, *ESR1*, *CYR61*, *MKI67*, *GRB7*, *CCND1*, *KRT19*, *RPLP0* and *SCUBE2* was found to be significantly (*p* < 0.001) more expressed in CTCs.

Another group [132] performed with the CellSearch^®^ system both the enumeration of CTCs, by immunostaining for cytokeratins and CD45, and the molecular analysis of cells bound to EpCAM-coated ferrofluids and retrieved from the CellSearch^®^ system cartridge. Gene expression profile of blood samples from metastatic breast cancer (61% positivity) and from healthy donors enriched for EpCAM+ cells by the CellSearch^®^ system were performed by RT-qPCR of a panel including 1 epithelial cell-specific and 22 breast-specific genes. Twelve genes were found uniquely detected in all patients’ samples—*TFF1*, *ERBB4*, *CEA*, *IGFBP5*, *MAGEA3*, *SCGB2A2*, *TNRC9*, *PIP*, *PGR*, *SERPINB5*, *SCGB1D2*, and *EGFR*—and the remaining genes were more expressed in patients than healthy donors. Among genes highly expressed in CTCs, 27 (33.8%) and 25 (31.3%) patients expressed trefoil factor 1 (*TFF1*) and mammaglobin, respectively, and *KRT19* detection correlated with CTC count. *BST1*, a leukocyte-specific marker, was expressed in the majority of samples of healthy donors and patients with metastatic breast cancer, a result consistent with the detection of contaminating leukocytes in the CTC-enriched population. 

In another work, cells belonging to the CD45 positive (CD45, CD34, CD73, CD90, CD105, CD235) and CD45 negative lineage cells, i.e., putative CTCs, were isolated by FACS from patients with metastatic breast cancer [133]. Transcriptome analysis performed by RNA-sequencing uncovered a series of 188 genes significantly differentially expressed in hematopoietic lineage-positive versus lineage-negative cells, which included *CAVIN2*, *ITGB3*, *LY6G6F*, *TUBB1*, *LTBP1*, and *TRIM58*. Some genes associated with epithelial cells, such as *EPCAM*, *TACSTD2*, *MUC2*, *KRT7*, *KRT8*, *KRT18*, and *KRT19*, and genes that are specific to mammary tissue, such as *LTF* and *CTTN*, were detected in the lineage-negative population.

A large panel including 85 mRNA transcripts of clinical significance in breast cancer and with expected relevance for CTC detection was identified in 2011, upon selection from gene expression data sets and considering the gene expression level reported in literature for both white blood cells and breast tumor tissues [134]. CTCs had been isolated with the CellSearch^®^ system from patients with metastatic breast cancer and profiled by RT-qPCR. Comparative analysis with CellSearch^®^-enriched blood samples from healthy donors revealed that 55 genes had significantly higher expression in patients with at least 5 CTCs. Unsupervised hierarchical clustering according to gene expression patterns of 55 mRNA showed that healthy donors and breast cancer patients without detectable CTCs clustered closely together and could be clearly separated from the breast cancer patients with detectable CTCs. The authors identified four mRNA clusters, including genes related to cell signaling, luminal cell type, epithelial- and CTC-specific markers, and cell cycle progression and proliferation.

A complete list of genes reported in breast cancer studies as more expressed in CTC-enriched/positive samples or cells expressing epithelial markers or negative for markers of the hematopoietic lineage, compared to matched leukocytes or samples with undetected CTCs or blood samples from healthy donors, is shown in Table 1.

### 5.2. Clinical Significance of EMT, Metastasis and Chemoresistance-Related Gene Expression in Circulating Breast Cancer Cells

Tumor tissue biopsies have been the standard method to characterize breast cancer thus far. However, they have some drawbacks due to invasiveness, difficulties in accessing metastatic sites, and limited representativeness of the disease’s spatial and temporal heterogeneity and evolution. During tumor initiation and progression, cancer cells become able to adapt to micro-environmental stressors, to tolerate therapeutic regimens, and to evolve towards more and more aggressive phenotypes. Much of this phenotypic progression finally leads to the establishment of metastatic lesions, a process that in carcinomas is mainly related to the activation of the EMT program [138]. EMT is a reversible morphogenetic process that takes place during development, fibrosis and cancer. It is now recognized that EMT does not only culminate in a complete switch toward a fully mesenchymal phenotype, rather, discrete states or a continuum of phenotypic states exist along the epithelial-to-mesenchymal (E-to-M) spectrum [139,140]. As a result, cells in EMT may enter a variety of intermediate states and display several degrees of epithelial and mesenchymal features. Importantly, a link between EMT, stemness and resistance to therapy has been postulated, raising questions about the implication of the CTC phenotype in the success of clinical trials and the reduced effectiveness of current therapies in impairing or eradicating metastases [141]. A common starting point in EMT is the downregulation of epithelial features in epithelial cells that progressively acquire mesenchymal-like phenotypes [142,143]. The transcriptional changes and the modulation of cell polarity-related proteins and transcription factors during EMT imply that techniques for CTC detection in breast cancer relying upon the expression of the epithelial cell adhesion molecule (EpCAM) may underestimate CTCs with EMT features, as also chemoresistant and metastasis-initiating CTCs. On the other hand, the reverse mesenchymal-to-epithelial transition (MET) process, which restores the epithelial state, is critical for the establishment of contacts with other structures and cells in the metastatic site [144]. However, how MET and the epithelial features of cancer cells contribute to metastasis remains relatively unknown. In 1988, expression of E-cadherin was found to induce MET in mouse sarcoma cells [145], and in 2010 it was reported that ectopic expression of E-cadherin in MDA-MB-231 cells resulted in morphological and functional reversion of the epithelial phenotype [146]. Recently, in breast cancer models, it was observed that while loss of the epithelial marker E-cadherin increases invasion and dissemination in vitro, cells with E-cadherin loss generate significantly less lung metastases in vivo, and E-cadherin promotes the metastasis of invasive ductal breast carcinoma by enhancing the survival of tumor cells [147].

EpCAM-dependent isolation methods, such as the CellSearch^®^ system, predominate in breast cancer CTC clinical studies. EpCAM is perhaps the best known epithelial cell adhesion molecule for the fact that it is expressed in the majority of human epithelial cancers, including colorectal, breast, gastric, prostate, ovarian, and lung cancer [148,149]. Selecting for EpCAM+ve CTCs only might miss the detection of other CTC populations in E-to-M transition states and the opportunity to obtain a full picture of the tumor plasticity and in response to therapy. In the large trial SWOG500 [150] a change in treatment protocol based on CTC status as assessed by EpCAM-dependent selection through the CellSearch^®^ system did not provide benefit for patients with unfavorable CTC count in the advanced stage setting. In 2013, the importance of detecting CTCs in EMT [151] to assess response to therapy in real-time was demonstrated. CTCs were captured by using EpCAM, EGFR, and HER2 as cell surface target proteins in combination with the microfluidic device ^Hb^CTC-Chip, they were characterized by in situ hybridization with RNA probes for epithelial markers (*CDH1*, *EPCAM*, *KRT5*, *KRT7*, *KRT8*, *KRT18*, and *KRT19*) and mesenchymal markers (*FN1*, *CDH2*, and *SERPINE1*), and were subsequently classified into five categories: purely epithelial (E), purely mesenchymal (M), or intermediate (E > M, E = M, and M > E). As two mesenchymal cells were detected in one of the five blood samples from healthy donors, the CTC positivity cut-off was set to five cells per 3 mL of blood and, on the basis of such a threshold, 17 out of 41 patients with metastatic breast cancer were called as CTC positive, and all 17 cases showed evidence of phenotypic changes in their CTCs. In particular, a large fraction of CTCs was either double E/M-positive or M-positive among the HER2-positive and triple-negative subtypes. The longitudinal monitoring of CTC features in ten patients showed that, after targeted therapy, responders had CTCs with an epithelial-like or intermediate phenotype, while CTCs from refractory patients were more numerous and retained or acquired an M phenotype at progression.

The limitation of EpCAM-dependent CTC detection seems to be particularly pronounced in early stage breast cancer. As discussed in previous paragraphs, seminal studies showed that positivity is generally lower than 30% before starting primary systemic therapy or before surgical intervention, but the sensitivity of the enrichment method can substantially increase when analyzing large volumes of blood, or when using antigen-independent size-based selection, or when screening the white blood cell fraction for the expression of epithelial and breast-cancer associated markers and the presence of HER2 amplification, reaching positivity higher than 70%. Additionally, it is important to consider that, by virtue of the plasticity of EMT, it is possible to find CTCs in intermediate or partial EMT state, detectable by EpCAM antibodies and expressing at transcriptional level genes related to the EMT process. In fact, in a case series of patients with metastatic breast cancer the expression of *TWIST1*, *AKT2* and *PIK3CA* in EpCAM-based enriched CTC samples was evaluated, and it was found that 62% of them were positive for at least one of the EMT-related markers [152]. In other studies, it was shown that pathways that can regulate the EMT process, such as phosphorylated EGFR, HIF1α, HER2 and PI3K/Akt signaling, are expressed or activated in CTCs of women with breast cancer [153,154]. Overall, these results corroborate experimental data showing that hematogenous dissemination is a step of the metastatic process that can occur early in breast cancer, and clearly indicate that improving the accuracy of CTC detection might open up a novel scenario within the context of breast cancer diagnosis and disease progression monitoring.

CTC positivity as assessed by gene expression analysis is generally determined through the detection of *KRT19* in cell samples enriched for CTCs by immunomagnetic capture with anti EpCAM and/or anti MUC-1 antibodies. In breast cancer, positivity for *KRT19* in the metastatic setting may range between 30 and 80%, whereas in patients with non-metastatic breast cancer, percentages are generally around 20%, although some studies reported higher values up to 60% [155,156,157]. CTC positivity by gene expression analysis in breast cancer is also assessed by means of RT-PCR for other epithelial markers and breast normal/tumor tissue-associated genes, such as *EPCAM*, *MUC1*, *SCGB2A2* and *ERBB2*, whose detection frequencies in non-metastatic compared to advanced disease case series are 0–19% versus 17%, 0–41% versus 10%, 11–14% versus 20–34%, 6–14% versus 10–20%, respectively, per each gene [124,135,155,156,158,159]. *TERT* and *VEGF* gene transcripts were also more frequently detected in patients with advanced compared to those with early stage disease, with percentages of 19% versus 10% and 35% versus 24%, respectively, per each gene [156,157]. In 2012, a study involving about 500 patients with non-metastatic breast cancer revealed that, although CTC positivity was 19% as assessed by detecting at least one epithelial and breast cancer-associated transcript among *EPCAM*, *MUC1* and *ERBB2*, 90% of samples expressed at least one EMT-related gene among *PIK3CA*, *AKT2* and *TWIST1* based on cut-off values set in a case series of 30 healthy donors, an 18% of cases negative for epithelial or breast cancer markers were positive for at least one of the EMT genes [158]. *TWIST1* detection frequencies are highly variable, ranging from 2.4 to 42% in patients with non-metastatic breast cancer compared to 0–39% in advanced disease, although a trend toward slightly greater percentages in early stage cases are generally observed [155,156,157]. Such results have suggested that the subsets of CTCs in full or intermediate EMT phase are highly represented in early stage breast cancer. By using immunostaining of cytological samples of PBMCs, vimentin- and Twist-expressing cytokeratin-positive CTCs were identified in over 70% of pre-selected patients with early stage breast cancer and showing 88% positivity to cytokeratin staining, and in all patients with metastatic disease [160]. In 2015, it was found that CTC enrichment through antibodies directed against HER2 and EGFR, whose signaling may be involved in the EMT program, in addition to EpCAM and MUC-1, improves CTC detection in breast cancer, shifting from 13% positivity when using EpCAM and MUC-1 to 47% through immunomagnetic binding of surface HER2 and EGFR; detection frequency increased 4-fold in early stage and 3-fold in advanced stage cases, and in the early stage setting such an increase was mainly due to positivity signals of EMT and stemness rather than epithelial and breast cancer cell-related gene transcripts [124].

The clinical significance of CTCs expressing EMT-related genes in early stage breast cancer has not been clarified yet. In some studies, no association was observed between EMT gene expression in CTCs and risk of disease progression or overall survival, neither when assessing CTCs before treatment nor during or after neoadjuvant therapy [135,158,159]. In previous works, after depleting the EpCAM+ and CD45+ cell populations from blood samples of patients with early stage breast cancers, overexpression of the EMT inducing transcription factors *TWIST1*, *SNAIL1*, *SLUG*, *ZEB1* and *FOXC2* was observed in a significantly higher fraction of patients receiving neoadjuvant therapy compared to those not candidate to multimodal treatment [161]. In another study including 427 primary breast cancer patients the same authors reported that EMT transcripts were detected in 18% of patients and that those with undetermined EMT genes had significantly longer disease-free survival than patients with detectable CTCs in EMT [162]. Additionally, in a case series of 100 early stage breast cancer, it was found that patients with EpCAM-enriched CTCs overexpressing *TWIST1* and showing a stem cell profile, defined as CD44^high^/CD24^−/low^ and ALDH1^high^/CD24^−/low^, had significantly reduced disease-free and overall survival [163]. Overall results of these studies highlight the importance of assessing EMT gene expression levels rather than just detecting them in order to obtain clinically useful CTC-related gene markers. Consistently, patients candidate to neoadjuvant therapy positive for CTCs with a high score, i.e., more than 3000 transcripts by digital droplet PCR, for a CTC-specific 17-gene signature, including breast lineage-specific genes, oncofetal antigens, epithelial-derived cytokines, transcripts highly expressed in breast cancer, implicated in endocrine signaling and resistance, as also in cancer growth and metastasis, had worse prognosis compared to those with lower scores at baseline [129].

Another crucial point to be considered in CTC gene expression studies is the role of the selected panel of genes in metastasis and in chemoresistance since clinically informative CTCs are expected to be endowed with pronounced cell fitness, in order to survive in foreign microenvironments, in addition to the acquisition of EMT, and MET, features. Recently, by comparing the gene expression profile of CTCs isolated from the MDA-MB-231 xenograft model with those of primary tumor nodules and metastases at lymph nodes and lungs, it was demonstrated that hematogenous dissemination is driven by a massive transcriptional reprogramming, and that genes up-regulated and down-regulated in CTCs compared to solid lesions are enriched in gene ontology terms related to cell adaptation and chromatin remodeling, respectively [135]. Knowledge was provided on the role of two genes, *FADS3* and *TFF3*, in cell migration, invasion, blood-borne dissemination and lung tissue colonization, and the analysis of a panel of transcripts derived from the signature of 192 genes up-regulated in CTCs, including *ADPRHL1*, *FCF1*, *ELF3*, *TFF1* and *TFF3*, was shown to increase 2-fold CTC detection in case series when at least one of the five genes was detected in EpCAM/MUC-1-enriched CTC samples, compared to epithelial, breast cancer and EMT-related markers, reaching positivities of 83% and 65% in patients with metastatic and those with non-metastatic breast cancer, respectively, and to predict disease recurrence in the neoadjuvant setting when detected before and during treatment [135]. Regarding the clinical significance of chemoresistance-related gene expression in CTCs, longitudinal analysis of blood samples enriched in CTCs by size-base separation through a microfiltration technique showed that positivity in the neoadjuvant setting, by assessing the expression of *CD24*, *CD44*, *CD45*, *CD68*, *KRT19*, *EPCAM*, *MUC1*, *MGB*, *ERBB2*, *ESR1* and *PGR*, was 80% at baseline and remained high, around 70%, at the end of treatment; interestingly, 13 out of 17 CTC-positive cases expressed *MRP1*, *MRP2*, *MRP4*, *MRP5*, *MRP7*, *MDR1* and *ERCC1* chemotherapy resistance-associated markers [164].

CTC count in the advanced stage has been confirmed as an independent prognostic factor. CTCs should be virtually detectable in all patients with metastatic disease if considering that the tumor has progressed to the ultimate step of the metastatic cascade and tends to shed malignant cells from the site of the secondary lesions. In this context, identifying a CTC-gene signature predictive of response to therapy is fundamental for implementing CTC molecular analysis to guide clinical decision during treatment. Epithelial and breast cancer-related genes failed to predict response to therapy and disease progression when assessed in EpCAM-based enriched CTCs in patients with metastatic breast cancer starting first-line cisplatin-based therapy or treated with anthracycline and taxane [135,159,165]. However, some genes peculiar to luminal breast cancer are associated to the site of metastasis or response to endocrine therapy, such as *TFF1*, a classical estrogen-regulated gene, whose expression in CTCs was found to be a strong predictor of bone metastasis [132]. Hormonal receptor status in CTCs might have a predictive role, as patients with high expression of estrogen receptor β gene *ESR2* in CTCs exhibited better response to hormonal treatment, and patients with ER-negative primary tumors and ER-positive CTCs had a longer median time to treatment switch compared to those with concordantly ER-negative CTCs [132,165]. Two 8-gene signatures able to predict response to therapy, and overall including *CXCL14*, *KRT7*, *KRT19*, *KRT81*, *PKP3*, *PTRF*, *TIMP3*, *LAD1*, *S100A16*, *FKBP10*, *TWIST1*, *PTRF*, *EEF1A2*, *PTPRK*, *EGFR* and *ERBB3* genes, were generated by comparing the CTC profile of poor and good responders following first line treatment with standard hormonal or chemotherapy or with aromatase inhibitors only [166,167]. Other CTC-detected genes involved in drug resistance, such as *MTOR*, *PIK3CA*, *ADAM17*, *ALDH2*, *TIMP1*, *PALB2* and *MYC*, in addition to overexpressed epithelial and EMT-related markers, such as EpCAM^high^, CD44^high^, and ALDH1^high^, may have prognostic significance or can be found more frequently expressed in poor responders [136,137,168,169,170].

**Figure 2:**
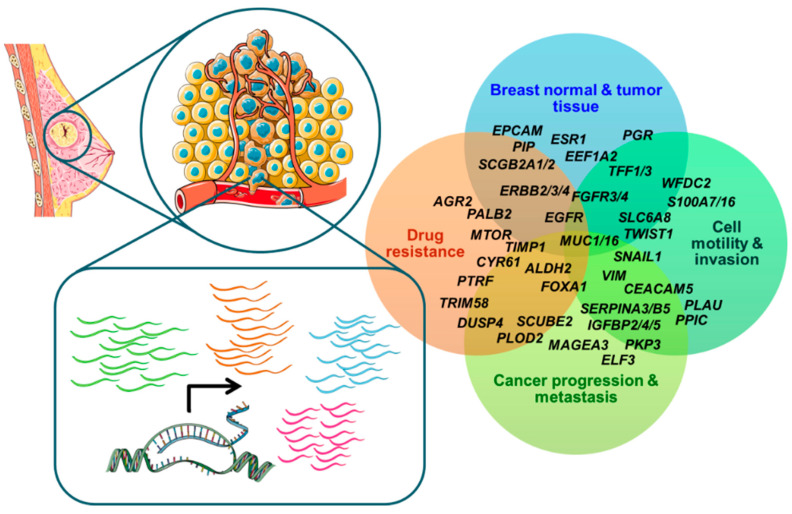
Breast cancer cell hematogenous dissemination is governed by a transcriptional reprogramming. Genes actively transcribed (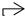) or modulated in circulating tumor cells in patients with early-stage or metastatic breast cancer are related to normal and tumor breast tissue development, epithelial-to-mesenchymal transition, cell motility, migration, invasion and metastatic ability, drug resistance and immune system evasion, tumor growth and progression. (Cartoon depicting breast cancer cell hematogenous dissemination and transcription process was adapted from templates available in smart.servier.com, accessed on 26 October 2022).

Breast cancer circulating cell-related gene signatures to early classify patients at higher risk of post-operative disease recurrence or to predict response to systemic therapy for metastatic disease have been identified and might have relevant roles as additional biomarkers for disease subtyping and designing tailored treatment protocols (Figure 2). However, the lack of uniformity in methods for sample processing and the existence of several subpopulations of CTCs, which can be missed based on the type of enrichment technique, are making the process of blood-test development a challenging goal. The advent of novel technologies for single cell retrieval from complex biological matrices, and their downstream molecular analysis, might impact future studies on CTC transcriptome analysis and its application in the clinical context.

### 5.3. Sources of Breast Cancer Molecular Signatures from Ex Vivo Expanded CTCs and Circulating Cell-Free Tumor-Derived Biomolecules

Molecular signatures discovered in in vitro cultured or in vivo injected CTCs, established to perform functional and pharmacological studies, and in cell-free DNA (cfDNA), which is largely regarded as a cellular component released by active secretion from tumor cells, including CTCs, and not only originating through apoptosis, necrosis, phagocytosis and other cell death processes, have provided additional insight into CTC heterogeneity and its clinical potential in breast cancer.

Considering that organotropism is a hallmark of breast cancer, CTCs are expected to comprise subpopulations of cells characterized by both gene expression patterns peculiar to the metastatic site and organ-specific mutational profiles [171]. In CTCs associated with brain metastases of breast cancer, for instance, a higher activation of Notch signaling and inflammatory and immunomodulatory networks that might be involved into immune evasion and mitotic reactivation compared to non-brain metastasis CTCs or CTCs associated to other metastatic sites was observed [172]. Moreover, the CTC population related to brain metastases showed a higher *SEMA4D* expression level compared with CTC lines with no brain tropism in immunocompromised mice, suggesting a role for semaphorin as a mediator of blood-brain barrier transmigration in CTCs [173]. By analyzing gene expression arrays of cell populations generated from CTC-derived xenograft models and online available databases, other authors have found a CTC-related liver metastasis-associated signature in triple-negative breast cancer [174]. An epithelial and luminal B subtype-related gene expression signature was observed in a CTC-derived breast cancer cell line established from a patient with metastatic ER+ breast cancer, resistant to endocrine therapy, both in adherence and non-adherence culture conditions [175]. A divergent gene expression profile was found in CTCs lines derived from patients with advanced breast cancer as they showed higher expression of genes related to stemness and mesenchymal phenotype when cultured under low-adherence conditions compared to both matched primary CTCs and CTCs with reduced capabilities to grow ex vivo [176].

Several groups have compared CTC enumeration with cfDNA detection in breast cancer, but data describing the complementarity and differences between cfDNA and CTC genomic DNA variants are still scarce. Druggable mutations detected by cfDNA analysis are generally found in *PIK3CA*, *ESR1*, *ERBB2*, *PTEN* and *AKT1* genes in patients with metastatic breast cancer [177]. Recently, a comprehensive analysis of variants of matched cfDNA and CTC samples by targeted deep sequencing in patients with hormone receptor-positive HER2− metastatic breast cancer revealed that information acquired from both analytes complement each other: 23 variants were common to both cfDNA and CTCs, whereas 104 and 14 were exclusively found in CTCs and cfDNA, respectively [178]. Overall, such findings indicate the existence of CTCs with clonal heterogeneity as a function of their proclivity to disseminate in one or another site and of their ability to survive as intact cells, elude the immune system or resist standard and targeted therapies, all features that are mirrored in the dynamic changes of each CTC transcriptome profile.

## 6. Challenges to the Integration of CTC Molecular Analysis in the Clinical Practice and Future Perspective

Although the detection of CTCs remains a challenge due to their rarity in the blood circulatory system compared other blood cell types, the proliferating number of studies currently ongoing indicates that scientists and clinicians can count on a plethora of technical approaches to perform CTC analysis. CTC enumeration definitively proved to be a strong predictor of worse outcome both in early and advanced stage breast cancer. Whether, by CTC counting and by analyzing CTC fluctuations, we will be able to guide the decision to shift toward a different treatment regimen or protocol, in addition to the early identification of patients with the highest risk of disease relapse or progression, is still a debated matter that deserves intensive and attentive investigation. Due to a certain degree of discordance in CTC positivity in breast cancer among several studies, especially if considering the early stage setting, and to the heterogeneity within the CTC population, further areas of particular interest are represented by the development of effective protocols for detecting all CTC subsets, in order to minimize possible biases introduced by the enrichment and selection process, and for unveiling the information harbored in the CTC transcriptome to take the maximum advantage of CTC characterization. Perhaps the greatest challenge to the development of CTC-based gene signatures useful for breast cancer diagnosis, prognosis and prediction of treatment response is related to breast cancer heterogeneity. Gene signatures traditionally derived from the analysis of the tumor bulk provide a snapshot of a tumor’s gene expression profile in a single time point. The leading determinants of the majority of signatures in breast cancer are proliferation, ER-status, and HER2-status, while genes related to angiogenesis and invasiveness are less represented. Moreover, the presence of subpopulations of tumor cells that differ in their genetic makeup, response to therapy and metastatic potential might be underestimated. Therefore, the process of establishing gene signatures is complex and caution must be taken when using primary tumor profiles for clinical decisions on systemic therapies to prevent or treat metastases. 

CTCs are generally regarded as a surrogate of a tumor biopsy, but it is hard that CTC analysis will be accepted as an alternative tool to catch tumor heterogeneity, as they represent a minimal fraction of the tumor and not all CTCs possess the same metastatic potential. However, the intrinsic characteristics of cancer cells are known and have been largely described thanks to decades of investigation focused on the pathogenesis of cancer, which provided clarity on the processes involved in neoplastic transformation, whereas defining the hallmarks of metastasis and dissecting the molecular events of each step of the metastatic process is a challenging goal due to the diversity of mechanisms involved and of routes of dissemination. Several gene expression patterns have been implicated in cancer metastasis and each step of the metastatic cascade might be mediated by different sets of genes. The expression of such genes can influence the proclivity of some cancer cells to intravasate and start a journey through the circulatory system. As clinical oncology progresses towards personalized cancer medicine, the need to understand the biology of metastasis becomes increasingly urgent.

Although CTC detection and molecular analysis protocols are not ready for a scale-up in large patient cohorts, single CTC transcriptomics has the potential to trace the plasticity of the true drivers of cancer progression and to profile a population of tumor cells with determinant role in therapy resistance. Moreover, comparison analysis between CTCs and primary tumor cells can highlight some of the features that could play a role in CTC ability to interact with other cells, intravasate, and survive in the blood microenvironment. Modulation of gene expression and shift toward different patterns proved to initiate such fundamental steps of the hematogenous dissemination process. Importantly, non-polyadenylated transcripts, such as small nucleolar RNAs, histone mRNAs, pre-mRNAs, and long noncoding RNAs, may play diverse regulatory roles in cancer in addition to messenger RNA, but the majority of single cell RNA sequencing data regards poly-A transcripts, thus limiting the ability to examine the whole tumor cell transcriptome. Importantly, the timing of blood sampling is expected to impact CTC detection and the level of information obtained from CTC molecular analysis, therefore blood sample comparisons over time and long-term CTC follow-up would deserve consideration for the design of future clinical studies. Finally, practitioners, patients, caregivers and families need to receive detailed guidelines for applying CTC-based tests in the clinical practice and information about access to screening programs and multigene testing. Cancer screening is expected to address disparities as patients should benefit from coverage of costs for blood tests, physician consultation and subsequent treatment. Healthcare and coverage policies are evolving to include specific cancer screening and molecular tests and to proactively integrate them into the oncology care continuum, but health insurance coverage and cost-effectiveness balance issues have not been fully addressed yet.

## 7. Conclusions

Gene expression profile analysis has been rapidly helping to solve the complexity of breast cancer, but the identification of new biomarkers to predict early disease relapse and detect chemoresistance timely as also the development of more effective therapies to increase current standards of care are still pressing clinical needs. Breast cancer cells are prone to disseminate during the first steps of tumor initiation; therefore, increasing knowledge on the mechanisms at the basis of hematogenous spread is a priority. An encouraging, although still not exhaustive, amount of data on CTC detection, enumeration and gene expression profile in breast cancer consolidates the existing rationale for implementing CTC analysis in the clinical routine. Experimental and clinical studies provided evidence that CTCs possess different transcriptome profiles compared to primary tumor cells, and that not all CTCs have the same metastatic ability. Therefore, merging findings coming from basic science with clinical observations is key for elucidating the biology of hematogenous dissemination. To date, transcriptomic profiling of CTCs has not reached its full potential due to the lack of protocols for catching different CTC subtypes and the technical limits of current single cell analysis pipelines. Through the investigation of the transcriptional changes that accompany and regulate cancer cell intravasation, survival and metastagenicity, and by comparing primary tumor and CTC-borne biomarkers, we could have access to another level of breast cancer heterogeneity and significantly contribute to biomarker development and drug discovery.

## Figures and Tables

**Table 1:** Gene transcripts for CTC detection in breast cancer.

Gene Abbreviation	Gene Function or Role in Cancer	Reference to CTC Studies
*ACTA2*	invasion	[134]
*ADPRHL1*	ADP-ribosylhydrolase	[135]
*AGR2*	cancer progression, chemoresistance	[129,131,134]
*ALDH2*	drug resistance, cancer stemness	[136]
*AR*	breast cancer development	[136]
*BCL11A*	tumorigenesis, metastasis	[136]
*CAVIN2*	tumor suppressor	[133]
*CCND1*	cell cycle	[131,134]
*CCNE2*	cell cycle	[127]
*CD24*	migration, invasion, anti-phagocytic	[134,137]
*CD44*	cell adhesion, migration, cancer stemness	[136]
*CEA*	cancer progression	[132]
*CEACAM5*	cell polarity, differentiation	[134]
*CEP55*	mitotic cytokinesis, development, cancer progression	[134]
*CLDN3*	cell adhesion	[134]
*CRABP2*	retinoic acid shuttling	[134]
*CRIPTO*	embryonic development, tumor growth	[136]
*CTTN*	cytoskeleton, cell adhesion structure	[134]
*CXCL13*	epithelial-derived cytokine	[129]
*CXCL14*	epithelial-derived cytokine	[129,134]
*CYR61*	chemoresistance	[131]
*DKFZp762E1312*	cell cycle	[127]
*DPP4*	metastasis	[128]
*DTL*	cell motility	[134]
*DTX3*	cell proliferation	[134]
*DUSP4*	chemoresistance	[134]
*EEF1A2*	breast cancer development	[134]
*EFHD1*	highly expressed in breast cancer	[129]
*EGFR*	tumor progression	[132]
*ELF3*	breast cancer progression	[135]
*EMP2*	cancer stemness, metastasis	[127]
*EPCAM*	cell adhesion, migration	[128,131,134,137]
*ERBB2*	cancer survival and progression	[131]
*ERBB3*	metastasis, therapy resistance	[134]
*ERBB4*	cancer progression, therapy resistance	[132,134]
*ESR1*	breast carcinogenesis	[128,131,134]
*FAT1*	cell adhesion, signaling	[129]
*FAT2*	cell adhesion, signaling	[129]
*FCF1*	pre-rRNA processing	[135]
*FEN1*	invasion, metastasis	[134]
*FKBP10*	migration, invasion	[134]
*FGFR3*	breast cancer development, endocrine resistance	[134]
*FGFR4*	luminal differentiation, metastasis	[134]
*FOXA1*	migration, invasion, endocrine resistance	[134]
*GRB7*	EGFR/ERBB2 signaling	[131]
*IGFBP2*	tumor growth	[134]
*IGFBP4*	tumor growth	[134]
*IGFBP5*	cell adhesion, survival	[132,134]
*IL17BR3*	endocrine resistance	[134]
*ITGA6*	cancer stemness, invasion	[134,136]
*ITGB3*	cell adhesion, signaling	[133]
*KIF11*	cell proliferation	[134]
*KPNA2*	cell proliferation	[134]
*KRT7*	invasion, metastasis	[134]
*KRT8*	regulation of invasion	[128]
*KRT16*	invasion, metastasis	[128]
*KRT17*	proliferation, invasion	[134]
*KRT18*	regulation of invasion	[134]
*KRT19*	cell proliferation	[128,131,134,137]
*LAD1*	breast cancer progression	[134]
*LTBP1*	metastasis	[133]
*LY6G6F*	breast cancer progression	[133]
*MAGEA3*	breast cancer progression	[132]
*MAL2*	breast cancer immune evasion	[127]
*MELK*	breast carcinogenesis	[134]
*MGP*	highly expressed in breast cancer	[129]
*MKI67*	cell proliferation	[131,134]
*MUC1*	cell survival, therapy resistance	[131,134]
*MUC16*	cancer growth and metastasis	[128,129]
*MUCL1*	cell proliferation	[134]
*PGR*	breast lineage specific	[128,129,132]
*PIP*	breast lineage specific	[129,132,134]
*PKP3*	breast cancer growth and progression	[134]
*PLAU*	motility, invasion	[134]
*PLOD2*	tumor progression, metastasis	[134]
*PPIC*	cell migration, invasion	[127]
*PRAME*	oncofetal antigens	[129]
*PTRF*	multidrug resistance	[134]
*S100A7*	cell survival, chemotaxis	[134]
*S100A16*	invasion	[134]
*SCGB1D2*	breast lineage specific, cell growth	[132,134]
*SCGB2A1*	breast lineage specific, cell growth	[129]
*SCGB2A2*	breast lineage specific, cell growth	[132,134]
*SCUBE2*	motility and invasion regulation	[131]
*SEPP1*	oxidative stress reduction	[134]
*SERPINA3*	EMT, invasion	[129]
*SERPINB5*	tumor growth and metastasis regulation	[132]
*SFRP1*	cellular signaling	[129]
*SFRP2*	cellular signaling	[129]
*SLC6A8*	invasion	[127]
*SNAIL1*	EMT inducer	[136]
*SPDEF*	pro- and anti-oncogenic among breast cancer subtypes	[134]
*TERT*	cancer development	[128]
*TFF1*	proliferation, migration	[131,132,134,135]
*TFF3*	migration, angiogenesis	[131,134,135]
*TIMP1*	cell survival, angiogenesis	[136]
*TIMP3*	cell growth, invasion, angiogenesis	[134]
*TM4SF13*	breast cancer growth regulation	[134]
*TMPRSS4*	cancer growth and metastasis	[129]
*TNRC9*	breast cancer progression	[132,134]
*TRIM58*	chemoresistance	[133]
*TUBB1*	β-tubulin	[133]
*TWIST1*	EMT inducer	[128]
*VIM*	cell motility	[136]
*WFDC2*	EMT inducer	[129]
